# Biosecurity practices on small-ruminant farms in five Turkish provinces: a cross-sectional survey with multiple correspondence analysis

**DOI:** 10.3389/fvets.2025.1677002

**Published:** 2025-11-17

**Authors:** Mafalda Pedro Mil-Homens, Ipek Keskin Fernandez Georges, Eran Raizman, Daniel Beltrán-Alcrudo, Alberto Allepuz

**Affiliations:** 1 Universitat Autònoma de Barcelona, Barcelona, Spain; 2 Food and Agriculture Organization of the United Nations (FAO), Regional Office for Europe and Central Asia, Budapest, Hungary

**Keywords:** biosecurity, small ruminants, health management, survey analysis, Türkiye

## Abstract

**Introduction:**

A cross-sectional biosecurity survey was conducted in Türkiye to assess practices in small ruminant farms across five provinces.

**Methods:**

A total of 364 breeders were interviewed on farm management, health practices, animal purchase and movement, and dead animal disposal. Breeders were selected based on small ruminant density, breed diversity, primary income source, presence of commercial enterprises, and branding potential. Among participants, 332 responses were eligible for analysis. Descriptive statistics and Multiple Correspondence Analysis (MCA) were used.

**Results:**

The most commonly administered vaccines were for sheep pox (193/332, 58%), foot-and-mouth disease (175/332, 53%), and brucellosis (129/332, 39%). About 58% (195/332) reported direct contact with ruminants from other herds, 30% (101/332) shared vehicles or equipment, 59% (196/332) attended live animal markets, and 99% (328/332) purchased animals, yet 67% (222/332) did not quarantine and only 14% (46/332) considered health status before purchase. Dead animals were buried (129/332, 39%) or fed to dogs (30/332, 9%). MCA indicated biosecurity improvements were needed across provinces, education levels, herd sizes, and production types.

**Discussion:**

The high proportion of direct contacts highlights the need for community-based interventions, shared quarantine facilities, physical barriers, and targeted training on disease recognition, disinfection, and record-keeping to strengthen herd health.

## 1 Introduction

In 2021, Türkiye’s sheep and goat population reached 57.5 million, marking a 6 percent increase from the previous year, with sheep accounting for approximately 80% of this total (1). Agriculture in Türkiye is predominantly based on small-scale enterprises, which present challenges for sectoral development. Türkiye’s small ruminant production largely relies on extensive farming systems, with most breeds being multipurpose (2).

Considering the important role played by small ruminant farming in Türkiye’s agriculture and its dependence on extensive farming practices, it is crucial to maintain their health and productivity, protecting the livelihoods of the farmers who rely on them (3, 4). Efficient disease prevention and control strategies are critical to the growth and sustainability of this sector (5), with biosecurity playing a central role (6). Despite increasing attention to livestock biosecurity, existing studies in Türkiye indicate that small ruminant farms frequently exhibit gaps in disease prevention and health-protection practices. For example, surveys in Balıkesir and Sakarya provinces revealed deficiencies in quarantine measures, water management, and neonatal care, highlighting the need for targeted training and improved farm management (7). In Yozgat province, traditional family-run sheep enterprises were found to have limited compliance with biosecurity standards, despite adequate shelter and welfare conditions (8). Similarly, a study in Niğde province in Türkiye highlighted a general lack of biosecurity implementation on small ruminant farms, raising concerns about disease prevention and control measures (9). Across these studies, it is suggested that many farms are interconnected through shared pastures, labor, and equipment, creating community-level pathways for disease transmission. Identifying patterns of farm management and biosecurity adoption is therefore critical. Multivariate approaches, such as Multiple Correspondence Analysis (MCA), can help classify farms according to their biosecurity practices, enabling targeted interventions and collective strategies to improve disease prevention (10).

In response to the limited evidence of biosecurity practices in Türkiye’s small ruminant farms, the FAO-Türkiye Partnership Programme on Food and Agriculture (FTPP II) launched the project “Improving efficiency of small ruminant production for reduction of GHG emission intensity” (GCP/SEC/014/TUR, funded by the Government of Türkiye). The primary aim of this survey was to assess the level of biosecurity implementation in small ruminant farms across different provinces of Türkiye and to identify factors influencing these practices. Specifically, the survey sought to answer the following questions: (1) What are the current biosecurity measures applied by small ruminant breeders? (2) How do farm characteristics and management practices influence biosecurity adoption? and (3) Which areas require targeted interventions to improve disease prevention and control?

The five provinces were selected to represent diverse production systems and geographic regions of Türkiye, based on criteria including the density of small ruminants, the presence of distinct breeds, the primary source of income, the existence of commercial enterprises, and the potential for branding of regional livestock products.

## 2 Materials and methods

### 2.1 Study design and population characteristics

**Figure 1:**
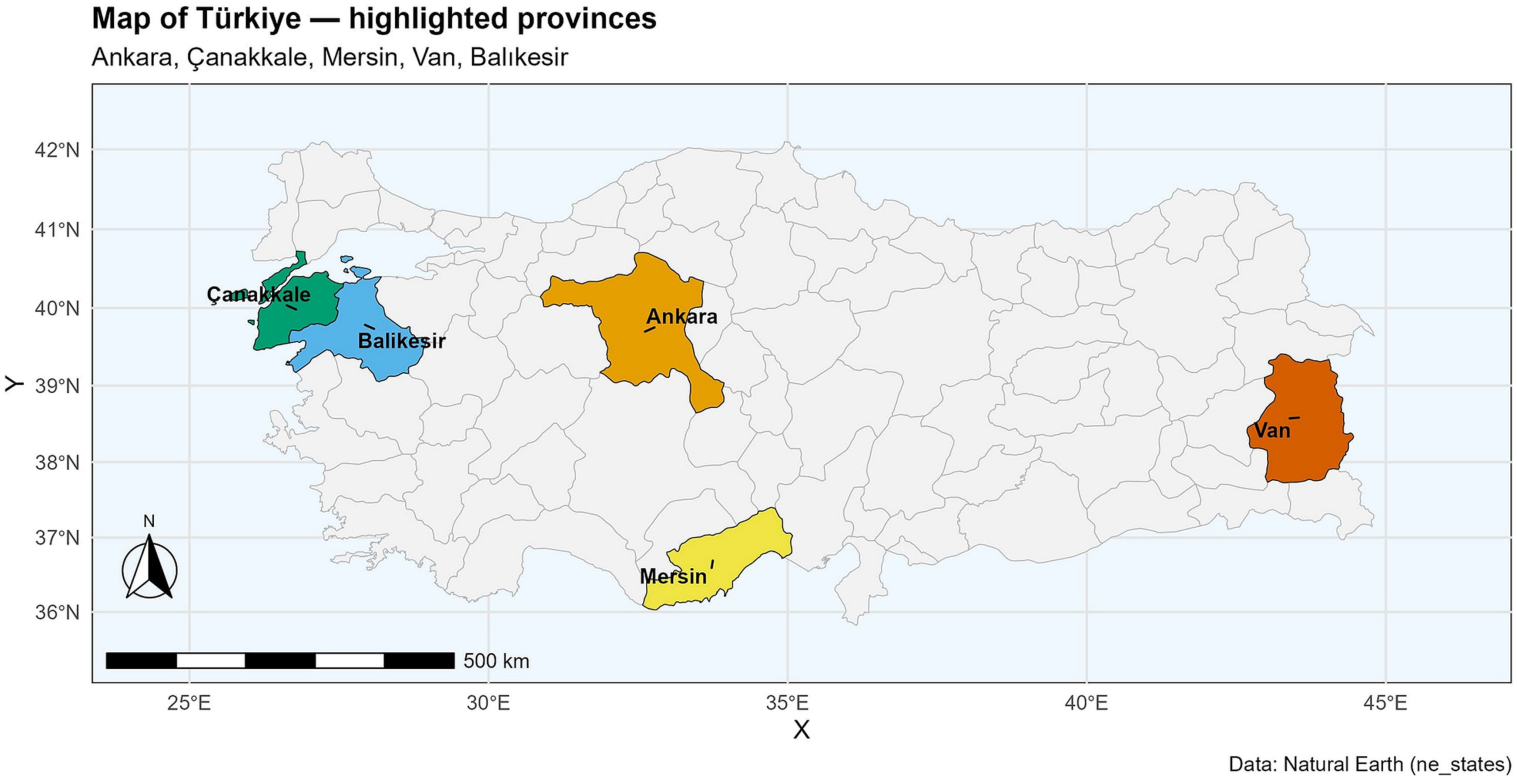
Map illustrating the five Türkiye provinces that were surveyed.

A cross-sectional survey was conducted between December 2022 and August 2023 in five provinces of Türkiye: Ankara, Balıkesir, Çanakkale, Mersin, and Van (Figure 1). These provinces were selected based on small ruminant density, the presence of distinct breeds, the primary source of income, the existence of commercial enterprises, and branding potential. Provincial directorates of the Ministry of Agriculture and Forestry assisted in identifying breeders. Participants were purposively selected from provincial lists to ensure representation of diverse production systems (sheep, goat, and mixed herds), herd sizes (small, medium, and large), and management practices. Inclusion criteria were breeders actively managing small ruminant herds during the study period and willing to participate in face-to-face interviews. Breeders were excluded if they were temporarily absent, had no active animals, or declined participation. Fieldwork was conducted by a team of seven trained interviewers, including in-depth interviews with 104 key informants at local and national levels to capture expert perspectives. Additionally, 12 focus group discussions were held with breeders and state veterinarians to validate observations and gain community-level insights. Semi-structured questionnaires were administered to 364 small ruminant breeders during farm visits. The herd size was categorized into small, medium, and large, with the number of animals for each herd size differing between regions (Table 1). Responses were recorded on paper and later digitized into Microsoft Excel. The survey consisted of eight sections. The first three focused on general information, farm management, and health management. The remaining five sections addressed risk factors for possible disease introduction and spread in the farms, including direct contact, indirect contact, live animal movements (such as purchases and sales), and dead animal management. The full questionnaire is available in Supplementary Material 1.

**Table 1:** Small ruminant herd size per each Turkish province analyzed.

Province	Small	Medium	Large	Non-response herd size
Ankara	0–250 (number of farms = 22)	250–750 (number of farms = 22)	≥750 (number of farms = 28)	Number of farms = 62
Çanakkale	0–70 (number of farms = 7)	70–100 (number of farms = 28)	≥100 (number of farms = 28)	Number of farms = 51
Balıkesir	0–70 (number of farms = 40)	70–250 (number of farms = 18)	≥250 (number of farms = 27)	Number of farms = 77
Mersin	0–70 (number of farms = 1)	70–100 (number of farms = 41)	≥100 (number of farms = 13)	Number of farms = 39
Van	0–70 (number of farms = 50)	70–150 (number of farms = 39)	≥150 (number of farms = 41)	Number of farms = 30

### 2.2 Data analysis

The analysis was done using R© Statistical Software (v4.4.0; R Core Team, 2024) and Microsoft Excel©. A descriptive analysis, including frequency tables, was done to characterize the biosecurity practices (see Supplementary Material 2).

Multiple Correspondence Analysis (MCA) is a technique used to analyze categorical data by reducing a large set of variables into a smaller number of components that capture the key patterns in the data (10, 11). As an extension of Correspondence Analysis (CA), MCA examines relationships between multiple categorical variables and can be viewed as a categorical counterpart to Principal Component Analysis (PCA) (10). MCA is applied to an indicator matrix, where each variable level is represented by binary values (0 or 1). It can also handle quantitative variables by recoding them into categorical bins. In MCA, each row has the same total, and the resulting distances between categories in a multidimensional space reflect their associations. Categories that frequently occur together are plotted closer, while those that rarely co-occur are placed farther apart. In this study, MCA was used to quantify categorical data and identify the dimensions that best distinguish between the different categories (11). Responses recorded as “missing” were excluded from the analysis, and no imputation was performed. The MCA was performed using the FactoMineR (12) and factoextra (13) packages. The analysis was conducted separately by province, education level, and herd size to explore group-level variation in biosecurity patterns. Eigenvalues and inertia were examined to determine the number of dimensions to retain, and the first two dimensions were used for graphical interpretation. For each group (province, education level, herd size), the mean coordinates of individual farms were computed to represent their average position in the MCA space. The most common answers for each biosecurity variable were summarized per group using frequency counts to aid interpretation. Clusters were visualized using two-dimensional MCA plots with ellipses showing the concentration of farms by group (province, education level, herd size). Categories located close to one another in the MCA biplots indicate biosecurity practices that frequently co-occurred.

### 2.3 Operational definitions

For the purpose of this study, key terms and variables were defined as follows:

- Quarantine: The practice of separating newly purchased or introduced animals from the resident herd for a minimum of 15 days to observe for clinical signs of disease before allowing contact with other animals.
- Health checks: Regular observation or veterinary examination of animals for signs of illness, including clinical examination and collection of samples and laboratory diagnostic testing.
- Direct contact: Direct physical interaction between animals from different herds, or wild and domestic animals.
- Indirect contact: Potential disease transmission through contaminated fomites, such as equipment, clothing, feed, or water sources, without physical contact between animals.
- Sharing vehicles/equipment: The use and sharing of transport vehicles (e.g., for animal movement) or farm equipment (e.g., feeding or cleaning tools) by more than one farm without proper cleaning and disinfection between uses.

### 2.4 Missing data management

Data completeness was evaluated prior to analysis. Responses with excessive missing information were excluded to ensure data quality. A threshold of 30% missing responses per individual questionnaire was pre-specified for exclusion. This value was selected to balance data integrity with sample retention, following the recommendations of Little and Rubin (14), who suggest that up to 20% of missing data is generally acceptable. No imputation methods or formal missing-data adjustments were applied to the retained dataset. Analyzes were performed on the available data after exclusion. For clarity in the text, total percentages are reported, while valid percentages are presented in tables to account for item-level missing responses.

## 3 Results

Due to a significant number of missing responses, and following the recommendations of Little and Rubin (14), data from 332 out of the 364 respondents (91%) were included in the final analysis.

### 3.1 General information

Among the respondents, 20% (67/332) were from Ankara, 24% (81/332) were from Balıkesir, 17% (57/332) from Çanakkale, 14% (47/332) from Mersin, and 24% (80/332) from Van. Most farmers were male (280/332, 86%), aged between 41 and 50 years (126/332, 38%), and had with primary school education. Of the 332 farmers, 267 (80%) provided shelter for the animals, used primarily during the winter at night. During the summer, animals were mostly kept in the pasture. Regarding herd composition, 23% (75/332) managed mixed herds (sheep and goat), 67% (221/332) managed sheep herds, and 11% (36/332) managed goat herds. In terms of herd size, 48% (160/332) were classified as large, 33% (108/332) as medium, 17% (57/332) as small, and 2% (7/332) did not have the herd size (see Supplementary Material 2).

### 3.2 Farm and health management

Concerning farm management, most chores on the farm were carried out by the husband, except for milking, which was primarily the wife’s responsibility (see Supplementary Material 2). Concerning health management, when animals required veterinary assistance, 68% (225/332) of farmers called the veterinarian. Sixty percent (200/332) reported following the vaccination and deworming calendar issued by authorities, with 98% (324/332) vaccinating the sheep, 91% (303/332) deworming their sheep, and 54% (180/332) also deworming their dogs. The three most administered vaccines were for sheep pox (58%; 193/332), foot-and-mouth disease (53%; 175/332), and brucellosis (39%, 129/332), all provided through government programs. Most farmers (56%, 186/332) viewed vaccination as the best disease prevention method. When animals got sick, 37% (124/332) of farmers treated them, with 90% (299/332) getting medication from a veterinarian. Around 39% (129/332) of farmers answered that they have lost below 10 sheep due to a disease in the past year, approximately 20% (65/332) mentioned they lost between 10 to 20 sheep, around 2% (7/332) mentioned they lost between 20 and 40 sheep, the same amount mentioned losing more than 40 (8/332) sheep, and the remaining did not answer. Concerning the cause of animal loss, 33% (109/332) identified it as being a nutritional or digestive problem, 9% (31/332) identified it as being an infectious disease, 8% (25/332) identified it as reproductive problems or mastitis, and the remaining identified other issues, such as parasites. Five percent (17/332) did not know the cause, and the remaining (42%, 140/332) did not answer the question (see Supplementary Material 2).

### 3.3 Direct and indirect contact with other herds

According to 56% (185/332) of farmers, animals had regular direct contact with domestic ruminants from other herds in pastures, and 27% (90/332) shared breeding areas. Concerning indirect contact, 46% (153/332) reported regularly sharing vehicles, equipment, and shepherds with other herds (Table 2).

**Table 2:** Frequency and percentage of practices for direct and indirect contact.

Practices	Answer	Frequency	Total percentage (%)^*^	Total confidence interval (%)	Valid percentage (%)^*^	Valid confidence interval (%)
Frequency of animals’ contact with sheep/goats from other herds in the pasture	Always	49	14.8	[11.3–19.0]	17.3	[13.3–22.2]
	Frequently	63	19.0	[15.1–23.5]	22.2	[17.8–27.5]
	Non-response	49	14.8	[11.3–19.0]	–	–
	Never	98	29.5	[24.9–34.6]	34.6	[29.3–40.3]
	Sometimes	73	22.0	[17.9–26.7]	25.8	[21.0–31.2]
Sharing common space with other breeders to breed the animals	Non-response	151	45.5	[40.2–50.9]	–	–
	No	91	27.4	[22.9–32.4]	50.3	[43.1–57.5]
	Yes	90	27.1	[22.6–32.1]	49.7	[42.5–56.9]
Sharing breeding space or contact between domestic animals in pasture	Non-response	47	14.2	[10.8–18.3]	–	–
	No	195	58.7	[53.3–63.9]	68.4	[62.8–73.5]
	Yes	90	27.1	[22.6–32.1]	31.6	[26.4–37.2]
Sharing vehicles or equipment with other breeders	Non-response	151	45.5	[40.2–50.9]	–	–
	No	80	24.1	[19.8–29.0]	44.2	[37.2–51.5]
	Yes	101	30.4	[25.7–35.6]	55.8	[48.5–62.8]
Common shepherd	Non-response	70	21.1	[17.0–25.8]	–	–
	No	129	38.9	[33.8–44.2]	49.2	[43.2–55.3]
	Yes	133	40.1	[34.9–45.4]	50.8	[44.7–56.8]
Sharing vehicles/equipment or a shepherd with other herds	No	179	53.9	[48.5–59.2]	53.9	[48.5–59.2]
	Yes	153	46.1	[40.8–51.5]	46.1	[40.8–51.5]

^*^Total Percentage—number of answers in the category divided by the total number of breeders.

^**^Valid Percentage—number of answers in the category divided by the total number of breeders minus the unanswered questions (non-response).

### 3.4 Animal movement

Animal trade was common, with 59% (196/332) of farmers taking their animals to live animal markets, and 99% (328/332) buying animals in the past 2 years, with only 14% (46/332) considering health status before purchasing animals. Of the 328 who bought animals, 67% did not implement quarantine. Among those who quarantined animals (104 breeders), 18% did not perform health checks. Concerning transportation during purchases, 44% (147/332) shared their vehicle or equipment with other farmers occasionally, and 11% (37/332) did so frequently. For animal selling, 56% (185/332) of farmers sold their animals to traders, while the rest sold directly to other farms, butchers, or slaughterhouses. The majority (34%; 114/332) sold their animals once a year (Table 3).

**Table 3:** Frequency and percentage of practices involved in animal selling and purchase.

Practices	Answer	Frequency	Total percentage (%)^*^	Total confidence interval (%)	Valid percentage (%)^**^	Valid confidence interval (%)
Selling animals at live animal markets/religious or cultural festivities	Yes	196	59.0	[54.7–64.1]	62.2	[56.8–67.3]
	No	119	35.8	[30.9–41.1]	37.8	[32.6–43.2]
	Non-response	17	5.1	[3.2–8.0]	–	–
Purchased animals in the past 2 years	No	4	1.2	[0.5–3.0]	1.2	[0.5–3.0]
	Yes	328	98.8	[96.9–99.5]	98.8	[96.9–99.5]
Ensure health on animal purchase	Health related	46	13.9	[10.6–18.0]	21.2	[16.3–27.1]
	Non-response	115	34.6	[29.7–39.9]	–	–
	Non-health related	171	51.5	[46.1–56.8]	78.9	[72.9–83.7]
Quarantine days	<15 days	40	12.1	[9.0–16.0]	12.1	[9.0–16.1]
	≥15 days	64	19.3	[15.4–23.9]	19.4	[15.5–24.0]
	Did_not_purchase	4	1.2	[0.5–3.0]	1.2	[0.4–3.1]
	Non-response	2	0.6	[0.2–2.1]	–	–
	No quarantine	222	66.9	[61.6–71.7]	67.2	[62.0–72.1]
Health checks are performed in quarantine	Did_not_purchase	4	1.2	[0.5–3.0]	1.2	[0.5–3.1]
	Non-response	1	0.3	[0.05–1.7]	–	–
	No	19	5.7	[3.7–8.9]	5.7	[3.7–8.8]
	No_quarantine	222	66.9	[61.6–71.7]	67.1	[61.8–71.9]
	Yes	86	25.9	[21.4–30.9]	26.0	[21.6–31.0]
Transport used for purchase	Non-response	49	14.8	[11.3–19.0]	–	–
	My car	137	41.3	[36.1–46.6]	48.4	[42.6–54.2]
	Other	146	44.0	[38.7–49.4]	51.6	[45.8–57.4]
Sharing vehicles/machinery with farms for animal purchase	Frequently	37	11.1	[8.2–15.0]	13.0	[9.6–17.4]
	Non-response	48	14.5	[11.1–18.6]	–	–
	Never	100	30.1	[25.4–35.3]	35.2	[29.9–40.9]
	Sometimes	147	44.3	[39.0–49.7]	51.8	[46.0–57.5]
Selling lambs	Another farmer	19	5.7	[3.7–8.8]	6.9	[4.5–10.6]
	Butcher	36	10.8	[7.9–14.6]	13.1	[9.6–17.7]
	Non-response	58	17.5	[13.8–21.9]	–	–
	Other	9	2.7	[1.4–5.0]	3.3	[1.7–6.1]
	Slaughterhouse	25	7.5	[5.2–10.9]	9.1	[6.3–13.1]
	Trader	185	55.7	[50.3–61.0]	67.5	[61.8–72.8]
Frequency of sale (number of times per year)	1	114	34.3	[29.4–39.6]	44.9	[38.9–51.0]
	2	65	19.6	[15.7–24.2]	25.6	[20.6–31.3]
	3	39	11.8	[8.7–15.7]	15.4	[11.4–20.3]
	4	23	6.9	[4.7–10.2]	9.1	[6.1–13.2]
	5	7	2.1	[1.0–4.3]	2.8	[1.3–5.6]
	6	4	1.2	[0.5–3.1]	1.5	[0.6–4.0]
	>6	1	0.3	[0.05–1.7]	0.4	[0.007–2.1]
	Often	1	0.3	[0.05–1.7]	0.4	[0.007–2.2]
	Non-response	78	23.5	[19.2–28.3]	–	–

^*^Total Percentage—number of answers in the category divided by the total number of breeders.

^**^Valid Percentage—number of answers in the category divided by the total number of breeders minus the unanswered questions (non-response).

### 3.5 Management of dead animals

Of the 209 farmers who responded, 62% buried carcasses, 4% fed them to dogs, 5% discarded them in pastures, and 2% reported other practices. In addition, from the 177 farmers who answered the question “Are there dead animals in the pasture?,” 11% answered “Yes.” Of the 282 farmers who responded to the question of dogs having access to dead animals or aborted materials, 60% answered “Yes.” These results can be observed in Table 4.

**Table 4:** Frequency and percentage of practices involved in dead animal management.

Practices	Answer	Frequency	Total percentage (%)^*^	Total confidence interval (%)	Valid percentage (%)^**^	Valid confidence interval (%)
Carcass disposal	Bury	129	38.9	[33.8–44.2]	61.7	[55.0–68.0]
	Give to the dog	30	9.0	[6.4–12.6]	14.4	[10.2–19.8]
	Non-response	123	37.1	[32.0–42.4]	–	–
	Other	17	5.1	[3.2–8.0]	8.1	[5.1–12.6]
	Pasture	33	9.9	[7.2–13.6]	15.8	[11.5–21.3]
Dead animals in the pastures	Non-response	155	46.7	[41.4–52.1]	–	–
	No	109	32.8	[28.0–38.1]	61.6	[54.2–68.4]
	Yes	68	20.5	[16.5–25.1]	38.4	[31.6–45.8]
Dogs’ access to offal from dead sheep/goats or aborted materials	Non-response	50	15.1	[11.6–19.3]	–	–
	No	114	34.3	[29.4–39.6]	40.4	[34.9–46.2]
	Yes	168	50.6	[45.2–55.9]	59.5	[53.8–65.1]

^*^Total Percentage—number of answers in the category divided by the total number of breeders.

^**^Valid Percentage—number of answers in the category divided by the total number of breeders minus the unanswered questions (non-response).

### 3.6 Multiple correspondence analysis (MCA)

Due to the number of missing answers, the MCA analysis was implemented by considering only the cells in the dataset that had observations to avoid clusters solely based on missing answers.

#### 3.6.1 Province clusters

**Figure 2:**
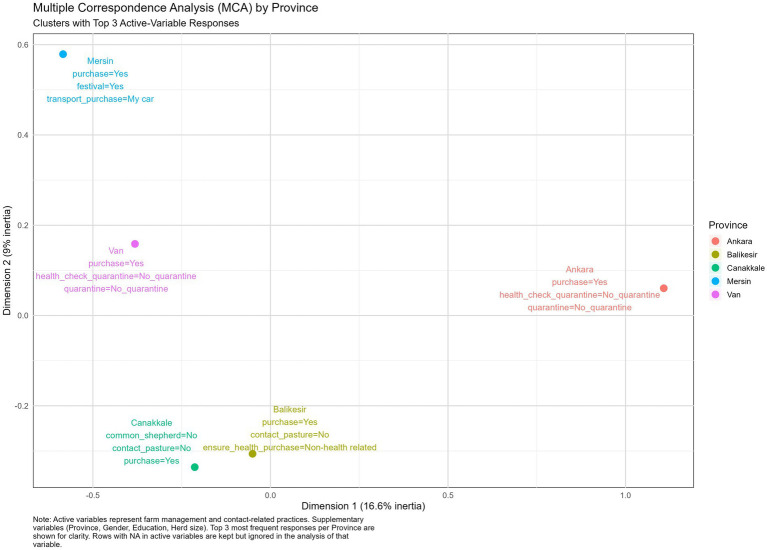
Multiple correspondence analysis illustrating the top three answers for the clusters of the five provinces surveyed.

The results from the MCA indicated both similarities and differences in biosecurity practices across the five Turkish provinces surveyed. Animal purchase was observed in all provinces, Ankara (a central province), Balıkesir and Çanakkale (western provinces), Van (an eastern province), and Mersin (a Mediterranean province), with Ankara, Van and Balıkesir not performing health checks before or after animal purchase. For Çanakkale, this province exhibited a pattern of not sharing pastures and not sharing shepherds with other herds (see Figure 2).

#### 3.6.2 Education clusters

**Figure 3:**
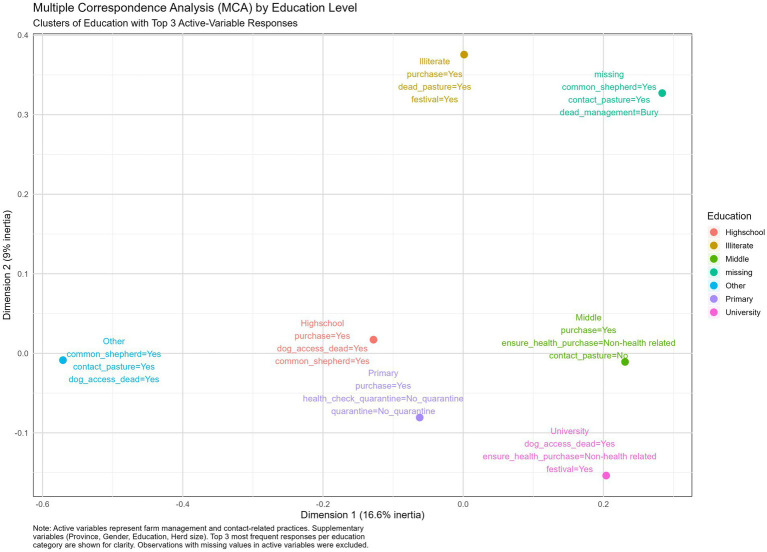
Multiple correspondence analysis illustrating the top three answers per education level cluster.

Regarding the education level, animal purchases happened in most education levels, with major gaps in biosecurity concerning the lack of health checks before purchase or during quarantine, and throwing dead animals into the pastures, and dogs having access to animal carcasses (Figure 3).

#### 3.6.3 Herd size and type of production system clusters

**Figure 4:**
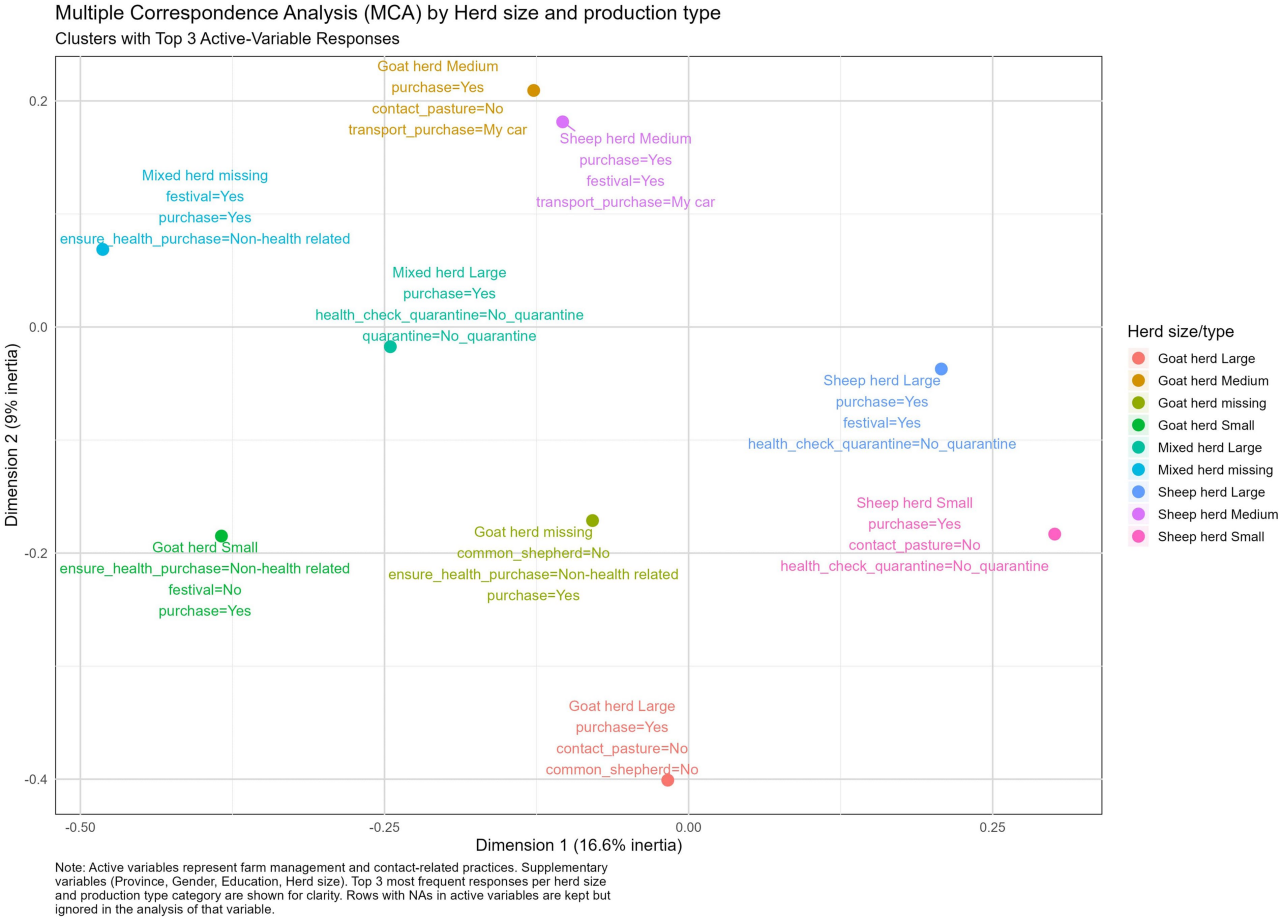
Multiple correspondence analysis illustrating the top three answers for herd size and type of production cluster.

Similar to what was observed for the provinces and education levels, the herd size and production type clusters also revealed that farmers purchased animals independently of herd size or production type. Issues concerning not implementing quarantine and not performing health checks were observed in three out of the nine clusters, and no contact in the pasture was observed in three out of the nine clusters (Figure 4).

## 4 Discussion

Biosecurity is a critical aspect of animal health management on small ruminant farms, particularly in countries like Türkiye, with its large population of sheep and goats (1, 15). Effective biosecurity measures are essential to prevent the introduction and spread of infectious diseases, ensuring both animal health and farm productivity. This study provides new insights into biosecurity practices among small ruminant farmers across five provinces in Türkiye. The findings indicate significant variation in practices related to animal movement, contact, vaccination, and dead animal disposal, all of which carry implications for disease transmission and control.

While this survey provided a broad picture, it is limited by self-reporting and the exclusion of farms with high missing data. Indeed, one of the main challenges encountered was a significant amount of missing data. The survey had 15% missing answers (7,161 out of 48,048 answers), with 9% of farmers being removed from the analysis for having more than 30% missing answers in their survey. Questions most frequently left unanswered were related to specific management practices and quantitative details, such as the use of protective clothing, delegation of animal care, and disease losses. These omissions may reflect a lack of awareness, reluctance to provide certain information, sensitivity of certain management-related questions, or incomplete knowledge regarding biosecurity protocols. The missing responses pose a risk of bias, potentially skewing the results and limiting the generalizability of the findings (16).

Most farm management tasks were carried out by men. However, milking was typically performed by women, posing an often overlooked high risk of contracting zoonoses such as brucellosis, which can be transmitted through direct contact with infected animals or their fluids. While the survey did not specifically address biosecurity measures during the milking process, it is important to highlight that raising awareness and promoting the use of gloves and hygiene measures should be a focus of health education campaigns to mitigate the risk of brucellosis transmission (4). In addition, it is crucial to also address other human exposure risks in farming tasks, such as parturition, and handling sick or dead animals (17).

Approximately 56% (185/332) of respondents reported that their animals had direct contact with animals from other herds, which poses a very high risk of disease introduction, as cross-contamination can occur through shared grazing areas, water sources, or direct physical contact. In addition to direct contact, indirect contact through shared vehicles and common shepherds was widely reported (46%; 153/332), also posing a risk, as pathogens can easily be transported on contaminated surfaces or equipment (3).

Such community-level practices, sharing pastures, equipment, and shepherds, or animal transport, amplify the potential for inter-herd transmission, effectively converting those communities into one single epidemiological unit. However, they might be essential for the subsistence of farming communities, helping reduce treatment or vaccination costs, sharing labor, and improving access to equipment (18, 19). Strengthening the sense of communal farming, on top of farm-level measures, could substantially improve outcomes. Providing proper biosecurity training and raising awareness among farmers on the consequences of the disease on the farming community is of the utmost importance to minimize risks while maintaining contact between herds and cultural traditions.

The observed high rate of purchasing animals without adequate health screening or quarantine measures underscores a major vulnerability to disease introduction into a herd. Proper health evaluation, along with an enforced quarantine period, is crucial for ensuring that new animals do not harbor infectious diseases (20). Nearly 99% of respondents reported purchasing animals in the past 2 years. Among these, only 46/332 (14.0%) considered the health status of the animals before purchase. Quarantine practices were limited: 40/332 (12.1%) of purchased animals were quarantined for less than 15 days, and 64/332 (19.3%) were quarantined for 15 days or more. Health checks during quarantine were performed in 64/332 (19.3%) of cases, while 19/332 (5.7%) quarantined animals did not receive health checks. The majority of farms (222/332; 66.9%) did not quarantine purchased animals at all. However, in rural areas of Türkiye, farmers are often compelled to buy animals due to economic pressures, which can limit the adoption of proper quarantine and health screening practices.

The management of dead animals is another key biosecurity concern. Of those farmers who answered (209 farmers), 62% indicated that they would bury dead animals on their premises. However, if not done properly, this practice can still pose health risks, as carcasses may be accessible to scavenging dogs or wild carnivores, facilitating the continuation of zoonotic disease cycles such as hydatidosis. In this cycle, dogs become infected after consuming infected offal or carcasses, subsequently shedding *Echinococcus* eggs in their feces, which contaminate pastures and can infect grazing livestock and humans. In humans, the formation of cysts takes place in different organs, leading to pain, anorexia, ascites, and neurologic symptoms, among others (21). Therefore, these practices are problematic because they allow for the possibility of disease transmission to other animals through direct contact or the consumption of a contaminated carcass (22). Nevertheless, in the absence of appropriate rendering services across the country, livestock owners are obliged to opt for these unsafe practices, in which case, it is important to guarantee that carcasses are buried in fenced or otherwise restricted areas, at a minimum depth of 1.5–2.0 meters with 0.9–1.2 meters of soil covering the carcass (23), and with the application of lime to accelerate decomposition and reduce scavenger access (22). Expanding community-level awareness and providing clear practical guidance on safe carcass disposal should therefore form a priority component of national biosecurity training programs.

The movement of animals to live animal markets, and religious or cultural festivities was common among the participants surveyed (196/332; 59%). While such events can be culturally significant, they also present a major biosecurity concern. The concentration of animals from different herds increases the likelihood of disease transmission, particularly if animals are not properly protected or screened for infectious agents or quarantined after joining the event (24, 25). The illegal movements of animals to supply the demand also impose a risk for disease introduction (26).

The results from the Multiple Correspondence Analysis revealed both overlapping patterns and differences in biosecurity practices across Turkish provinces, education levels, herd sizes, and production types. Despite forming clusters based on regional, educational, or operational variables, fundamental weaknesses in biosecurity practices persisted across all groups. This highlights that to address the vulnerabilities in biosecurity implementation, practical biosecurity training and biosecurity awareness should be enforced at a wider level, and should not be targeted to specific provinces, education levels, herd sizes, or production types. This study supports the need for participatory training approaches. Farmer Field Schools (FFS), which emphasize hands-on experimentation, critical thinking, and collective decision-making, may offer an effective platform for promoting farm biosecurity. Through such models, farmers can observe the impact of good practices on their own or neighbors’ farms, increasing acceptance and adoption, i.e., a participatory, group-based approach to learning where farmers meet regularly during livestock season to observe, discuss, and experiment with farming practices directly in a farm setting, emphasizing learning by doing, critical thinking, and collective decision-making (27, 28).

Additionally, the survey did not explore certain important biosecurity practices in detail, such as measures taken during milking, parturition, and the disinfection of vehicles or equipment between herds. Furthermore, information on the tests implemented during quarantine, the isolation of sick animals, and the frequency of contact between herds was not sufficiently addressed. Not covering these aspects in depth was primarily due to limitations in interview duration. Since the questionnaire also included questions related to production as part of a broader value chain analysis, it was necessary to limit its overall length. In practice, lengthy interviews can reduce both the accuracy and engagement of respondents, particularly given the time constraints and competing priorities of livestock owners. Experience suggests that respondents’ focus tends to diminish after approximately 45 min, which can compromise data quality in extended surveys. Future studies should aim to examine these areas more thoroughly to provide a comprehensive assessment of biosecurity practices.

Biosecurity remains a critical yet under-implemented component of small ruminant health management in Türkiye. This study reveals widespread gaps in quarantine, vaccination, and carcass disposal practices, alongside substantial inter-herd contact through shared spaces and resources. These patterns highlight systemic vulnerabilities that compromise both animal and public health, but also opportunities for intervention.

Effective solutions will require context-specific and community-driven approaches, mostly related to raising awareness and training. Participatory models such as Farmer Field Schools can help embed biosecurity knowledge into routine farm decision-making. Strengthening veterinary outreach, peer learning, and locally adapted guidance will be essential to improve compliance and uptake. Suggestions for improving biosecurity are described in Supplementary Material 3.

This study has several limitations that should be acknowledged. First, the data relied on self-reported information, which may be subject to recall bias or social desirability bias, as respondents might have over-or under-reported certain biosecurity practices. Second, non-response patterns were observed, particularly for questions involving specific management or economic practices, which may reflect either reluctance to disclose details or limited familiarity with formal biosecurity procedures. To preserve data quality, respondents with more than 30% missing answers were excluded from the analysis, which could introduce selection bias if the excluded farms differed systematically from those retained. Although this approach improved internal consistency, it may slightly limit generalizability to all small ruminant farms in Türkiye. Future research should aim to strengthen data completeness through simplified digital questionnaires, direct observation of farm practices, or triangulation with veterinary and administrative records. Incorporating mixed-method approaches, including observational audits and participatory validation, would also help to better capture real-world implementation of biosecurity practices.

## 5 Conclusion

In conclusion, this study demonstrates that there remains considerable scope to strengthen biosecurity implementation on small ruminant farms in Türkiye. Frequent interactions among breeders and their herds highlight the importance of coordinated, community-level biosecurity approaches rather than farm-level interventions alone. The persistence of biosecurity gaps across provinces, regardless of education level, herd size, or production system, indicates the need for a national, structured approach to improvement. To address these deficiencies effectively, policy frameworks should prioritize the development and dissemination of practical biosecurity guidance materials, supported by farmer field schools and on-farm demonstration programs. These platforms can serve as effective delivery mechanisms to translate technical standards into everyday practice, enhance breeder engagement, and support the long-term sustainability of national animal health strategies.

## Data Availability

The datasets presented in this article are not readily available because they are held by the Food and Agriculture Organization of the United Nations. Requests to access the datasets should be directed to the corresponding author.

## References

[1] USDA. Turkey: Livestock and Products Annual | USDA Foreign Agricultural Service (2022). Available online at: https://www.fas.usda.gov/data/turkey-livestock-and-products-annual-5 (accessed February 27, 2025).

[2] Ocak S Davran MK Güney O. Small ruminant production in Turkey: highlighting in goat production. Trop Anim Health Prod. (2010) 42:155–9. doi: 10.1007/s11250-009-9402-z, PMID: 19597751

[3] Wrathall AE Simmons HA Bowles DJ Jones S. Biosecurity strategies for conserving valuable livestock genetic resources. Reprod Fertil Dev. (2004) 16:103–12. doi: 10.10371/RD03083, PMID: 14972108

[4] Bayne JE Waters KM. Biosecurity for reproductive disease prevention in sheep and goats. Vet Clin North Am Food Anim Pract. (2025) 41:71–82. doi: 10.1016/j.cvfa.2024.11.009, PMID: 39741070

[5] Mutua EN Bett BK Bukachi SA Estambale BA Nyamongo IK. From policy to practice: an assessment of biosecurity practices in cattle, sheep and goats production, marketing and slaughter in Baringo County, Kenya. PLoS One. (2022) 17:e0266449. doi: 10.1371/journal.pone.0266449, PMID: 35390055 PMC8989345

[6] Saegerman C Parisi G Niemi J Humblet M-F Ron-Román J Souley Kouato B . Evaluation survey on agreement with existing definitions of biosecurity with a focus on livestock. Animals. (2023) 13:1518. doi: 10.3390/ani13091518, PMID: 37174555 PMC10177301

[7] Altinöz T Danyer E Gündoğan TT Keser O Öklen SB Bilal T. A cross-sectional study on animal nutrition and management evaluation of selected small ruminant enterprises in Sakarya and Balıkesir. J Ist Vet Sci. (2024) 8:321–34. Available at: https://dergipark.org.tr/en/pub/http-www-jivs-net/issue/89492/1605281

[8] Aslan A Tüfekci̇ H. Structural features, biosecurity and animal welfare assessment in sheep farms in Yozgat Province, Türkiye. J Hellenic Vet Med Soc. (2024) 75:8229–40. doi: 10.12681/jhvms.36157

[9] Şen ÖT Durmuş M Koluman N. Examination of structural characteristics and biosecurity of sheep farms in Niğde province. Turk J Agric Food Sci Technol. (2023) 11:1847–54. doi: 10.24925/turjaf.v11i10.1847-1854.6127

[10] Mori Y Kuroda M Makino N. Multiple correspondence analysis In: Mori Y Kuroda M Makino N, editors. Nonlinear principal component analysis and its applications. Singapore: Springer (2016). 21–8. doi: 10.1007/978-981-10-0159-8

[11] Abdi H Valentin D. Multiple correspondence analysis. Encycl Meas Stat. (2007) 2:651–7. Available at: https://personal.utdallas.edu/~herve/Abdi-MCA2007-pretty.pdf

[12] Husson F Josse J Le S Mazet J. FactoMineR: multivariate exploratory data analysis and data mining. CRAN. (2025). doi: 10.32614/CRAN.package.FactoMineR,

[13] Kassambara A Mundt F. Factoextra: extract and visualize the results of multivariate data analyses. Vienna: (2020).

[14] Little RJA Rubin DB. Statistical analysis with missing data. Hoboken: John Wiley & Sons, Ltd (2002). 24-40 p.

[15] Food and Agriculture Organization of the United Nations. (2025). United Nations data: Sheep and Goat Meat. FAOSTAT. Available at: https://data.un.org/Data.aspx?q=Meat%2C+turkey&d=FAO&f=itemCode%3A1807%3BcountryCode%3A223

[16] Ayilara OF Zhang L Sajobi TT Sawatzky R Bohm E Lix LM. Impact of missing data on bias and precision when estimating change in patient-reported outcomes from a clinical registry. Health Qual Life Outcomes. (2019) 17:106. doi: 10.1186/s12955-019-1181-2, PMID: 31221151 PMC6585083

[17] Şahin S. Personal biosecurity measures followed by ruminant veterinarians and farmers in the country of Georgia, Master’s degree thesis in Zoonoses and one health. Barcelona: Universitat Autônoma de Barcelona (2024).

[18] Wolff C Abigaba S Sternberg Lewerin S. Ugandan cattle farmers’ perceived needs of disease prevention and strategies to improve biosecurity. BMC Vet Res. (2019) 15:208. doi: 10.1186/s12917-019-1961-2, PMID: 31226988 PMC6588948

[19] Moje N Waktole H Kassahun R Megersa B Chomen MT Leta S . Status of animal health biosecurity measures of dairy farms in urban and peri-urban areas of Central Ethiopia. Front Vet Sci. (2023) 10:10. doi: 10.3389/fvets.2023.1086702, PMID: 37065239 PMC10090322

[20] Hersom M Irsik M Thrift T. Biosecurity and biological risk management for livestock enterprises. (2008). Ask IFAS - Powered by EDIS. Available online at: https://edis.ifas.ufl.edu/publication/AN194 (accessed February 28, 2025).

[21] Arminanzas C Gutiérrez-Cuadra M Carmen Farinas M. Hidatidosis: aspectos epidemiológicos, clínicos, diagnósticos y terapéuticos. Rev Esp Quimioter. (2015) 28:116–24. 26032995

[22] Gwyther CL Williams AP Golyshin PN Edwards-Jones G Jones DL. The environmental and biosecurity characteristics of livestock carcass disposal methods: a review. Waste Manag. (2011) 31:767–78. doi: 10.1016/j.wasman.2010.12.005, PMID: 21216585

[23] Sander JE Warbington MC Myers LM. Selected methods of animal carcass disposal. Javma. (2002) 220:1003–5. doi: 10.2460/javma.2002.220.1003, PMID: 12420777

[24] Brookes VJ Wismandanu O Sudarnika E Roby JA Hayes L Ward MP . Live wildlife trade in markets – a scoping review to inform risk assessment of emerging infectious diseases. Sci. Total Environ. (2021):2021.09.13.21263377. doi: 10.1101/2021.09.13.21263377,

[25] Beverelli C Rohit T. Global livestock trade and infectious diseases. Fiesole: European University Institute (2023).

[26] De Clercq K Cetre-Sossah C Métras R. Mision of the community veterinary emergency team to Bulgaria (16–21 July 2018). Bulgaria: PPR (2018).

[27] FAO F and AO of the UN. Farmer field schools: guidance document. Rome: FAO (2016).

[28] FAO F and AO of the UN. Livestock farmer field schools – Facilitator’s guide. Rome: FAO (2022).

