# Supplementary material for: Biosecurity practices on small-ruminant farms in five Turkish provinces: a cross-sectional survey with multiple correspondence analysis
Source: Front Vet Sci. 2025 Nov 17;12:1677002. doi: 10.3389/fvets.2025.1677002 (PMC12665762; doi:10.3389/fvets.2025.1677002)
Supplement: Supplementary file 1 [file Supplementary_file_1.docx]

**
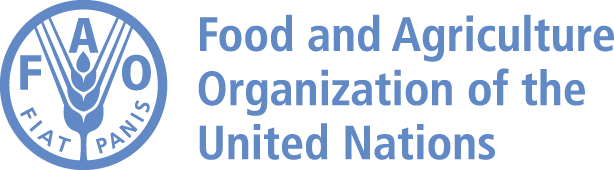
** Date:

No:

Name of the Enumerator:

Signature of Enumerator:

**VALUE CHAIN ANALYSIS**

**FARMER QUESTIONNAIRE**

Province / District: Village:

# 1. Demographic

1.Name and Surname (Optional):

2. Age:

1) 18-30 2) 31-40 3) 41-50 4) 51-60 5) Over 60

3. Gender:

1) Male 2) Female

4. Household Size:

1) Single-head 2) 1-2 3) 3-4 4) 5-6 5) Over 6

5. Education: 1) Illiterate 2) Primary school 3) Secondary school 4) Highschool

5) University 6) Other

**2. OWNERSHIP of LIVESTOCK**

6. What is your present herd size of sheep/lamb and rams? ______ (number)

1) Small 2) Medium 3) Large

7. What is your present herd size of goat? ______ (number)

1) Small 2) Medium 3) Large

8. How has the size of your herd changed over the last 3 years?

1) Increased 2) Decreased 3) Constant

8.1 What has been the reason for the change/ no change in your herd size? ________________________________

**3. HOUSING AND BREED MANAGEMENT**

9. Where do you keep your sheep?

9.1 Winter/Day

1. Pasture 2) Closed shed 3) Other ________________________
   1. Winter/Night
2. Pasture 2) Closed shed 3) Other ________________________
   1. Summer/Day
3. Pasture 2) Closed shed 3) Other________________

9.4 Summer/Night

1) Pasture 2) Closed shed 3) Other (Specify) __________________

10. Do you keep rams in your herd for the purpose of breeding?

1) Yes 2) No

10.1. Number of rams and how often are the rams included in the herd.

11. Do you purchase high quality rams for the purpose of breeding?

1) Yes 2) No

11.1 Which breed(s) of ram do you prefer for breeding and why? _____________________

12. Do you use a common shepherd?

1) Yes 2) No

13. On the pastures around the village, how frequently do your animals have contact with sheep/goats from other herds of the same village?

1) Never 2) Sometimes 3) Frequently 4) Always

14. What do you use to feed your animal?

14.1. What do you use to feed your animal in the Winter?

14.2. What do you use to feed your animal in the Summer?

15. Where and how do you meet your animal’s water needs?

**4. HEALTH MANAGEMENT**

16. Do you get your sheep vaccinated?

1) Yes 2) No

16.1 How many times in the last year did you get your sheep vaccinated? _______ (number)

16.2. Do you know the diseases for which the sheep is vaccinated?

1) Yes 2) No

16.3 If yes, Please Inform the name of the diseases (vaccination) ___________________________________

16.4 If you are not getting your sheep vaccinated then why not?

17. Is deworming done for your sheep?

1) Yes 2) No

17.1 If deworming is done, who is the service provider?

1) Local government veterinary service 2) Private veterinarian 3) Self 4) Other (specify)_________

17.2 If deworming is not done, then why not? _________________________________________________

18. Who do you contact for medical help for the sheep?

1) Local government veterinary service 2) Private veterinarian 3) Self 4) Other (specify)__________

19. What do you do when your animal is sick?

20. Where do you get the medicines if prescribed by the veterinarian?

1) From the veterinarian 2) Animal drug store 3) Local drug store 4) Not available 5) Other (specify)

21. How many sheep did you lose to diseases in the last one year? ________ (number)

21. How many goats did you lose to diseases in the last one year? ________ (number)

21.1 Can you specify the diseases/illnesses through which sheep are lost? (Names of the diseases or symptoms)

21.2 What do you do when your animal died?

22. Did you purchase sheep/goats in the last two years?

1) Yes 2) No

22.1 If yes, please complete the following tables (for the last 2 years):

|  | Number of sheep/goats | Number of times |
| --- | --- | --- |
| From others farms in the same village |  |  |
| From villages within the same province |  |  |
| From villages in other provinces |  |  |
| From other countries |  |  |

22.2 When purchasing sheep or goats, how do you ensure the health of the animals? ___________

22.3 When purchasing sheep or goats, do you isolate (quarantine) them on arrival from the rest of the animals of the herd?

1) Yes 2) No ________

22.3.1 If yes, 1. For how many days? :___________

22.3.2 If yes, 2. Do you perform any tests to check the health of the animals?

1) Yes 2) No

23. When purchasing sheep or goats, which kind of transport do you use?

1) Own vehicle 2) Other:__________

23.1 Do you share vehicles/machinery with farms from the same / other villages?

1) Never 2) Sometimes 3) Frequently

24. How do you maintain shelters?

1) Yes (and how many times) _______ 2) No (why?)

25. Do you regularly deworm the dogs that have contact with the animals from your herd?

1) Yes 2) No

26. Can those dogs have access and eat the offal from dead sheep/goats or aborted materials?

1) Never 2) Sometimes 3) Frequently 4) Always

27. In your opinion, what are the three most important measures to prevent the disease?

5. **Costs**

28. Can you specify the costs below?

| **Costs** | **Amount (TL)** |
| --- | --- |
| a.Feed ( per month) |  |
| b. Pasture rent ( per month)  c. Shepherd (per month / one shepherd) __________ |  |
| d. Veterinary services in a year ( per sheep) |  |
| e. Transportation (per month / milk and animal transport) |  |
| f. Medicine (per year) |  |
| g. Other items _______ |  |

**6. Meat - Marketing**

29. Who do you sell the lambs to? (circle all those which apply)

| **No** | **Buyer** | **Number of lambs sold in a year** | **Frequency of sale (number of times in a year)** | **Average age at the time of sale** | **Average weight at the time of sale (live Weight)** | **Price per sheep**  **Received (for live animal/TL)** |
| --- | --- | --- | --- | --- | --- | --- |
|  | Trader |  |  |  |  |  |
|  | Slaughterhouse |  |  |  |  |  |
|  | Butcher |  |  |  |  |  |
|  | Another farmer |  |  |  |  |  |
|  | Other (specify) |  |  |  |  |  |

29.1. Number of lambs sold in a year

29.2. Frequency of sales (number of times in a year)

29.3. Average age at the time of sale

29.4. Average weight at the time of sale

29.5. Price per sheep received

30. How many sheep do you sell in a single transaction? ________ (number)

31. Do you get any advance payment from any buyer?

1) Yes 2) No

32. Do you sell your sheep particularly at any festival?

1) Yes 2) No

33. What has been the overall trend in prices you have received for sale of sheep over the last 3 years? ____________

**7. Wool – Goat Hair / Sales and Marketing**

34. Is wool shearing done by you or by somebody else?

1) Myself 2) Somebody else

35. Is shearing of wool done manually or by machine?

1) Manually 2) By machine

36. Do you follow the basic hygiene measures for shearing? ___________________

37. Do you sell wool / hair goat?

1) Yes 2) No

37.1 If yes, who are you selling the wool to? ______________________

37.2 How much wool do you sell in a year? ________ kg

37.3 At what price is wool sold? ______ kg / TL

37.4 Do you do any kind of processing before sale of wool?

1) Yes 2) No

37.5 What has been the overall trend in prices you have received for sale of wool over the last 3 years?

37.6 If you are not able to sell wool, what do you do with the wool?

1) Throw away 2) Give away to relatives/friends 3) Burn it 4) Other (specify) _____________

**8. Milk / Production and Sale**

38. Do you milk your sheep on a regular basis?

1) Yes 2) No

38.1 What is the lactation period of your sheep? ______ (months)

38.2 What is the average milk production in a day from one lactating sheep? _______ (Milli Liter)

38.3 From your herd, how much milk do you obtain in total in: month: _____ (litres) year: _____ (litres)

38.4 Who takes your milk?

1) Union 2) Dairy farm 3) Neighbour 4) I don’t sell 5) Other ___________

38.5 How much do you sell a liter of milk? _______________TL/lt

39. Do you produce cheese from the sheep milk obtained?

1) Yes (What kind of cheese) 2) No

39.1 How much sheep cheese do you produce and sell in: month: __________kg year: _________kg

39.2 Who do you sell the cheese to?

1) Do not sell 2) Union 3) Local dairy 4) Nearby households 6) Other ________

39.3 What is the price received for 1 kg of cheese? ____________ TL/kg

40. In what form do you consume the milk you obtain from your sheep at home?

1) Yes _____________ 2) No

41. What has been the overall trend in prices you have received for sale of milk and cheese over the last 3 years?

1) Increasing 2) Decreasing 3) Constant

42. Approximately how many hours of family labor is required for care of sheep herd in 1 day? ________ hrs

| **Inputs -Services** | **Who takes the decision? (Code A)** | **Who is the responsible person in the family for these**  1. Man 2. Woman 3. Boy Child 4. Girl Child 5. Hired labour, 6. Any other (Specify) | **No. of hours required per day** |
| --- | --- | --- | --- |
| 42.1 Buying lamb |  |  |  |
| 42.2 Buying sheep (rams for the purpose of breeding) |  |  |  |
| 42.3 Shelter management |  |  |  |
| 42.4 Collecting feed and fodder |  |  |  |
| 42.5 Feeding the sheep (at home) |  |  |  |
| 42.6 Grazing outdoors/ pastures |  |  |  |
| 42.7 Health services |  |  |  |
| 42.8 Care of pregnant sheep |  |  |  |
| 42.9 Milking |  |  |  |
| 42.10 Sale of sheep |  |  |  |

43. What common issues you discuss on and take a decision with your family?

44. Do you get information regarding technical aspects of sheep rearing from any source?

1) Yes 2) No

45. Have you been provided with a vaccination and deworming calendar by the service provider?

1) Yes 2) No

46. Are you a member of farmer/breeder union?

1) Yes 2) No

47. How far is the nearest government veterinary point located from your farm? ____ km

48. Do you share a common space with other breeders to breed your animals?

49. Do you share any vehicles or equipment with other breeds?

50. Are your herd and wild animals in contact in the pasture?

51. Are there dead animals in the pasture?

52. How frequently do you consult with veterinarians per year?

53. Do you use special clothing and shoes to visit your animals?

54. Do you let him take care of the animals when you have visitors?
