# Supplementary material for: Biosecurity practices on small-ruminant farms in five Turkish provinces: a cross-sectional survey with multiple correspondence analysis
Source: Front Vet Sci. 2025 Nov 17;12:1677002. doi: 10.3389/fvets.2025.1677002 (PMC12665762; doi:10.3389/fvets.2025.1677002)
Supplement: Supplementary file 3 [file Supplementary_file_3.docx]

**Supplementary material 3. Biosecurity improvement measures**

**General farm management**

| **Risk** | **Measure** |
| --- | --- |
| Milking animals | When milking occurs, the person aiding should wear gloves, wash and disinfect the hands before and after, and avoid touching his/her eyes, nose and mouth during the process. The udder should also be cleaned and disinfected before the milking starts. If a machine is used for milking, the milking unit should be cleaned and disinfected after each use, and the rubber or silicone should be changed when there are signs of disintegration. |
| Lambing and kidding | The animal going into labor should be separated from the remaining animals. Gloves should be worn when aiding the animal in labor. Hands should be washed and disinfected before and after aiding the animal in labor. If the lamb or kid is born dead or if the animal has an abortion, it should be examined by a veterinarian and/or tested to determine the cause. The placenta and aborted fetus should not be fed to other domestic animals on the farm and should be destroyed. |
| Visitors | The use of personal protective equipment should be mandatory for all farm visitors. Farm visitors should have a place to change clothes and boots, and wash hands. |
| Water usage | The water used for consumption and cleaning should be clean and should be subjected to microbiological analysis at least once a year. |
| Contact with neighboring farms | Farms and grazing areas should be delimited by fences, and these should be well maintained to avoid contact between animals from different herds. If this is not possible, the animals should be vaccinated and dewormed before going to the shared areas. |

**General health management**

| **Risk** | **Measure** |
| --- | --- |
| Parasites and vectors | Implementing deworming in all domestic animals on the farm should be done regularly. Adding traps for vectors should be done, and areas with stagnated water should be removed. |
| Sick animals | Sick animals should be separated from the other animals in the herd, in a way that direct contact with healthy animals is avoided. Sick animals should be taken care of after all the other animals in the herd and specific personal protective equipment (PPE, i.e. gloves, boots, clothes, mask, goggles) should be used. Hands and boots should be washed after taking care of the animals. A veterinarian should be consulted. |

**Direct contact**

| **Risk** | **Measure** |
| --- | --- |
| Sharing pastures | The contact between animals of different herds in the pasture should be avoided. If this is not possible, then it should be guaranteed that the animals in the different herds are vaccinated and dewormed before going to the pasture. A community-based coordinated approach for health management should be explored with other farmers using the same pastures. |
| Sharing breeding space | Sharing the breeding space between different herds should be avoided, if that is not possible, then it should be guaranteed that animals are dewormed and vaccinated. A community-based coordinated approach for health management should be explored with other farmers using the same pastures. |

**Indirect contact**

| **Risk** | **Measure** |
| --- | --- |
| Sharing vehicles and equipment with other herds | Ideally, there should not be an exchange of vehicles, tools and equipment with other herds. If not possible, then the vehicles and the equipment should be cleaned and disinfected before and after each use. A community-based coordinated approach for health management should be explored with other farmers using the same pastures. |
| Sharing the same shepherd | Ideally, there should be one exclusive shepherd for each herd. If not possible, it should be guaranteed that the animals from the different herds the shepherd is working with are vaccinated and dewormed. A community-based coordinated approach for health management should be explored with other farmers using the same pastures. |

**Animal movements**

| **Risk** | **Measure** |
| --- | --- |
| Taking animals to live animal markets or festivities | If the animals that are taken to live animal markets or religious or cultural festivities do not return to the farm, then only health checks before traveling should be done to guarantee that the animals will not be spreading disease at the festival. If the animals return to the farm, then they should be put in quarantine and health checks should be performed. |
| Purchasing animals | When buying animals, these should originate from a herd that has the same health status as the herd that is purchasing, and health checks should be requested by the purchaser to the seller or done in the quarantine period at the farm. Laboratory tests may be conducted. |
| Quarantine | A quarantine period should be implemented when animals are purchased and when they return to the farm after being taken to markets or festivals. The quarantine pen should be kept far from the other animals in the herd. There should not be any contact between the animals in quarantine and those in the herd. The quarantine period should be at least one month, and health checks should be implemented. Animals in quarantine should be dealt with after the other animals in the herd and using PPE. Additionally, hands and boots should be washed after taking care of the animals. The quarantine pen should be washed and disinfected after the animals leave. |
| Sharing transportation for animal purchase | The vehicles should be cleaned and disinfected after every animal movement. When unloading animals to the farm, the vehicles should be kept far from the other animals on the farm, preferentially unloading the animals to the quarantine pen. |

**Selling animals**

| **Risk** | **Measure** |
| --- | --- |
| Selling animals at festivals | When selling animals at live animal markets or religious or cultural festivities, health checks should be performed before taking the animals to guarantee that the animals are healthy and will not spread disease. |

**Dead animal management**

| **Risk** | **Measure** |
| --- | --- |
| Carcass management | Carcasses should be destroyed using appropriate protocols far from the other animals in the flock and enclosed in an area where other domestic or wild animals cannot access the carcasses. The pavement should be impermeable to facilitate cleaning and disinfection. When dealing with carcasses, personal protective equipment should be used, and hands should be washed after taking care of the carcass. All the equipment for the carcass should be cleaned and disinfected. |
| Contact of domestic and wild animals with carcass | Carcasses should not be left/thrown in the pasture or fed to the dogs or other domestic animals. Carcasses should be enclosed in an area where other domestic or wild animals cannot access the carcasses. |
